# Successful hepatic resection for invasive *Klebsiella pneumoniae* large multiloculated liver abscesses with percutaneous drainage failure: A case report

**DOI:** 10.3389/fmed.2022.1092879

**Published:** 2023-01-06

**Authors:** Hiroyuki Nojima, Hiroaki Shimizu, Takashi Murakami, Masato Yamazaki, Kazuto Yamazaki, Seiya Suzuki, Kiyohiko Shuto, Chihiro Kosugi, Akihiro Usui, Keiji Koda

**Affiliations:** ^1^Department of Surgery, Teikyo University Chiba Medical Center, Ichihara, Japan; ^2^Department of Pathology, Teikyo University Chiba Medical Center, Ichihara, Japan

**Keywords:** *Klebsiella*-associated multiloculated liver abscess, percutaneous drainage failure, K1 serotype, hyper mucus discharge, *K. pneumoniae*

## Abstract

**Background:**

Invasive *Klebsiella*-associated liver abscesses can progress rapidly and cause severe metastatic infections such as meningitis and hydrocephalus, which are associated with high morbidity and mortality. In patients with large multiloculated liver abscesses after failure of percutaneous drainage, rapid diagnosis of the abscess followed by hepatic resection is necessary for early recovery and to prevent severe secondary metastatic complications.

**Case presentation:**

An 84-year-old woman with a large liver abscess and in septic shock was transferred to our hospital. Abdominal CT showed multiloculated liver abscesses 15 cm in diameter in the right lobe of the liver. We first performed percutaneous liver abscess drainage. The patient was managed in the intensive care unit, as well as treated with intravenous administration of meropenem followed by cefozopran according to the antibiogram. *Klebsiella pneumoniae* with invasive infection was confirmed by a string test in an isolated colony of *K. pneumoniae*; the K1 serotype with the *rmpA* and *magA* genes was determined by polymerase chain reaction and Sanger sequencing. Additional percutaneous liver abscess drainage was performed due to initial inadequate drainage. Although the abscess had shrunk to a diameter of 8 cm after drainage in 4 weeks, the patient recovered from sepsis, but still had low-grade fever (occasionally 38°C) and continued to have symptoms of chronic inflammation with persistent hyper mucus discharge from the liver abscess. Surgical resection was chosen to prevent prolonged hospitalization and ensure early recovery. A right posterior sectionectomy of the liver, including liver abscess, was performed. The post-operative course was uneventful, with no complications, and she was discharged after 18 days. There were no signs of abscess recurrence 1 year after surgery.

**Conclusion:**

We present a case of successful hepatic resection after percutaneous drainage failure in a patient with invasive *K. pneumoniae* multiloculated liver abscess.

## 1. Introduction

*Klebsiella pneumoniae* is the most common causative organism of bacterial liver abscesses and is classified into two groups, classical *K. pneumoniae* and hypervirulent *K. pneumoniae* (1). Hypervirulent *K. pneumoniae* was first identified in Taiwan in 1986 as a liver abscess associated with septic endophthalmitis in young and immunocompetent hosts (2). Hypervirulent strains have a higher potential to resist phagocytosis and metastasize to distant sites forming highly mucoid colonies with capsule polysaccharide, leading to a hypermucoviscous colony phenotype. Accordingly, it is also called hypermucoviscous *K. pneumoniae* (1). Hypermucoviscosity has been mostly associated with serotype K1, followed by serotype K2, and is associated with the hypervirulence phenotype of *Klebsiella* regulated by the mucoviscosity-associated gene A (magA) and regulator of mucoid phenotype A (rmpA) (3). *Klebsiella*-associated invasive liver abscess syndrome with the hypermucoviscous phenotype can progress rapidly and cause severe metastatic infections such as meningitis and hydrocephalus, which are associated with high morbidity and mortality (4, 5). A rapid diagnosis followed by treatment, such as percutaneous drainage and antibiotic therapy, is the standard for improving patient outcomes with hypermucoviscous *K. pneumoniae* (6). For large multiloculated abscesses with percutaneous drainage failure, surgical resection is necessary for early recovery and to prevent severe secondary metastatic lesions. Herein, we present a case of successful hepatic resection in a patient with an invasive *K. pneumoniae* multiloculated drainage-resistant liver abscess.

## 2. Case description

**FIGURE 1:**
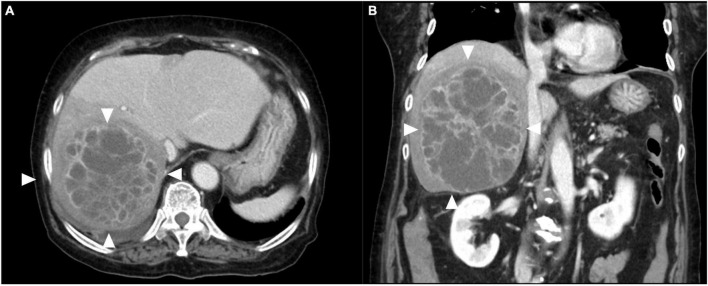
Contrast-enhanced computed tomography images of the liver. **(A)** Multiloculated liver abscess (white arrows) occupying the right lobes of the liver extending across the right hepatic vein. **(B)** Coronal image revealing multiloculated liver abscess (white arrows) 15 cm in diameter.

An 84-year-old woman, weighing 54 kg with a height of 150 cm, was urgently admitted to the previous hospital with a diagnosis of liver abscess. Antimicrobial agents for 1 week did not improve symptoms at the previous hospital, and she was transferred to our hospital with septic shock. The patient had an implant stem after a left femur fracture and a history of asthma. There was no history of diabetes or cancer, and no history of smoking, drinking alcohol, or drug allergies. On arrival at our hospital, her initial vital signs were as follows: body temperature, 38°C; heart rate, 122 beats/min; blood pressure, 64/50 mmHg; respiratory rate, 26 breaths/min; and oxygen saturation, 91%. Her blood test revealed an increased ammonia level of 127 μg/dl, decreased serum albumin level of 1.5 g/dl, and a bilirubin level of 0.8 mg/dl. Her white blood cell count was 18200/μL, PT-INR was 1.54, d-dimer was 27.4 ng/mL, and FDP was 32.3 U/mL. Her disseminated intravascular coagulation (DIC) score was 5 points. Abdominal computed tomography showed a multiloculated liver abscess 15 cm in diameter in the right lobes of the liver extending across the right hepatic vein (Figure 1). Extrahepatic infections, such as infections of the lungs and brain, were not detected.

**FIGURE 2:**
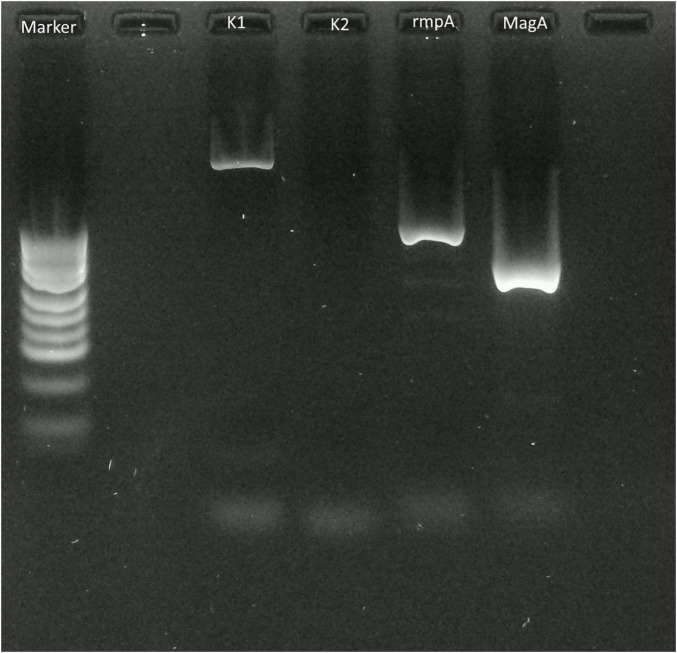
Polymerase chain reaction (PCR) applied to the pus of the liver abscess. PCR with extracted DNA from the pus of the abscess revealed amplification of the *Klebsiella pneumoniae* serotype K1 specific locus *(wzyKPK1), rmpA, and magA* fragments that were detected by electrophoresis on 2.0% agarose.

**FIGURE 3:**
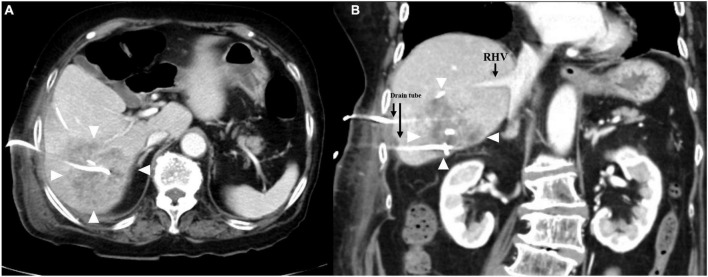
Contrast-enhanced computed tomography images of the liver. **(A)** Right hepatic vein separated from the multiloculated liver abscess (white arrows). **(B)** Coronal image revealing multiloculated liver abscess (white arrows) with drainage tubes 8 cm in diameter.

First, we performed percutaneous liver abscess drainage with two drainage tubes. Following transfer to the intensive care unit, intravenous meropenem followed by cefozopran was administered, according to the antibiogram. *K. pneumoniae* with invasive infection was confirmed by a string test in an isolated colony of *K. pneumonia*e, and invasive *K. pneumoniae* large liver abscess was diagnosed. We carried out polymerase chain reaction (PCR) with DNA from the aspirated fluid to detect the presence of hypermucoviscous *K. pneumoniae* as previously described (7). The PCR determined the amplification of specific genes for serotype K1, a regulator of mucoid phenotype A (rmpA), and mucoid associated gene A (magA), which was confirmed by Sanger sequencing (Figure 2). The additional abscess drainage was performed 1 week after the initial drain placement due to inadequate drainage, and finally, five drainage tubes were placed into the multiloculated liver abscess (Supplementary Figure 1). Although the abscess in the posterior segments of the liver had shrunk to a diameter of 8 cm at 4 weeks after drainage, the patient recovered from sepsis, but still had low-grade fever (occasionally 38°C), an elevated white blood cell count of 10,300/μL, C-reactive protein level of 6 U/L (Supplementary Figure 2), and continued to have symptoms of a chronic inflammation with persistent hyper mucus discharge from the liver abscess *via* five drainage tubes (Figure 3). We also checked for other causes of fever such as pneumonia and urinary tract infection, but no other infection related to fever was identified other than the liver abscess. We decided that further drainage treatment would be insufficient due to the multiloculated liver abscess with hyper mucus components and prolonged hospitalization with her advanced age. After explaining the patient’s condition and treatment options, a right posterior sectionectomy of the liver, including hepatic abscess, was performed. Although the abscess was not perforated, severe adhesions between the liver abscess and the right diaphragm were observed. The abscess was close to the root of Glisson’s sheath of the right posterior section; therefore, the right posterior sectionectomy of the liver, including the liver abscess, was performed (Supplementary Figure 3).

The operative time was 4 h and 48 min, and the total blood loss was 723 ml. The resected right posterior section of the liver revealed a multiloculated and necrotic liver abscess. Histological examination showed a pyogenic liver abscess with areas of necrosis, inflammatory granulation, and a few fluid components, composed of a chronic inflammatory infiltrate consisting of lymphocytes, epithelioid macrophages, eosinophils, and neutrophils. The adjacent hepatocytes appeared atrophic (Supplementary Figure 4). The post-operative course was uneventful, with no complications. The patient was discharged on post-operative day 18. Follow-up examinations using ultrasonography showed no signs of abscess recurrence 1 year after surgery.

## 3. Discussion

Liver abscesses can be divided into bacterial and amoebic liver abscesses; most of them are bacterial in origin, with *K. pneumoniae* reported to be the most common causative organism in bacterial liver abscesses (8). Invasive liver abscess syndrome caused by *K. pneumoniae* has been reported in Taiwan since the 1980s (2). *K. pneumoniae* is a mucoid-producing bacterium, and serotype K1 is the most common mucoid-producing strain of *K. pneumoniae*, followed by serotype K2 (3). In particular, *magA* has been identified as a gene associated with liver abscesses and metastatic lesions in K1 strains (3). Microbiological definitions of invasive liver abscess syndrome are described as *K pneumoniae* liver abscess caused by the K1 or K2 serotype (3). According to previous reports, some mucoid-producing strains of the genus *Klebsiella* are highly viscous, resistant to phagocytosis, and can spread hematogenously, resulting in secondary abscesses such as meningitis, hydrocephalus, endophthalmitis, and metastatic lung lesions (4, 5). Among these metastatic infections, endophthalmitis is the most serious complication and a poor prognostic factor for visual acuity (8). Rapid and accurate diagnosis of *K. pneumoniae*, such as the string test, and identification of the pathogen by metagenomic next-generation sequencing, is the key to start appropriate treatment immediately (9, 10). In our case, the patient had a positive string test, and PCR revealed amplification of the *K. pneumoniae* serotype K1 specific locus (*wzyKPK1*), *rmpA*, and *magA* fragments. Invasive liver abscess syndrome involving K1 strains is associated with an absence of immunodeficiency disorders; however, recent studies have shown that diabetes mellitus is recognized as an important risk factor for the development of the disease, as well as a poor prognostic factor for visual acuity (11). The frequency of spontaneous liver abscess rupture is as low as 3.8% (12). However, liver abscesses larger than 8 cm tend to rupture easily because of the high pressure of the contents, and ruptures presenting with peritonitis require emergency surgery (13, 14). In addition, an abscess larger than 6 cm is reported to be a significant independent risk factor for metastatic infections (15). Currently, combined antibiotic therapy based on the antibiogram and percutaneous catheter drainage of liver abscesses is the first choice of treatment for liver abscesses (16). Although many treatment strategies have been proposed for pyogenic liver abscesses, the indications for hepatic resection have not been systematically studied (17, 18). Surgical resection should be considered when there is no clinical improvement with antimicrobial therapy and percutaneous drainage, or if the liver abscess ruptures (19–21). Regarding multiloculated liver abscesses, the failure rate of percutaneous catheter drainage is high (22). Previous studies suggested that large multiloculated abscesses should be treated surgically (23). For large multiple liver abscesses with hyper mucus components, surgical resection should be performed if percutaneous drainage is inadequate, which may lead to improved prognosis and earlier recovery (24). In a report of 10 liver resections for *K. pneumoniae* liver abscess, including our case, 8 cases were multiloculated liver abscesses, 4 cases were operated due to failure of percutaneous abscess drainage, and 2 cases were due to abscess perforation. The post-operative course was reported to be uneventful in all cases (Table 1) (13, 19, 25–28). Regarding to the recurrence rate of liver abscess, the K1 serotype is known to be an important risk factor that influences liver abscess recurrence (29). This case report have some limitations. This was just a single case report, further accumulation of cases is necessary to establish the best therapeutic strategy for invasive *K. pneumoniae* liver abscess.

**TABLE 1:** Reported cases of hepatectomy for *Klebsiella pneumoniae* abscesses.

Cases	Age (year)	Sex	Abscess	Surgery	Reason for surgical treatment	Outcome
Shiba et al. (28)	84	Female	A gas-containing abscess (S2/3)	Lateral segmentectomy	Ruptured gas-containing liver abscess	Discharged 31 days after surgery
Morii et al. (13)	69	Female	A multiloculated abscess more than 10 cm in diameter (S7/8)	Partial segment S7/8 resection	Failure of antibiotic therapy and abscess perforation	Discharged 8 weeks after surgery
Maybury et al. (27)	74	Female	A multiloculated abscess (S2/3)	Left lateral sectionectomy	Failure of abscess drainage	Good recovery following surgery
Maybury et al. (27)	51	Male	A multiloculated abscess (S2)	Partial segment 2 resection	Failure of antibiotic therapy	Good recovery following surgery
Chan et al. (19)	41	Female	A multiloculated abscess 10 cm in diameter (S6/7)	Segment 6/7 resection	Failure of antibiotic therapy and abscess drainage	Discharged 6 weeks after surgery
Pais-Costa et al. (29)	54	Female	A multiloculated abscess 12 cm in diameter (Rt lobe)	Right hepatectomy	–	Discharged 23 days after surgery
Pais-Costa et al. (29)	66	Male	A multiloculated abscess 20 cm in diameter (Rt lobe)	Right hepatectomy	–	Discharged 25 days after surgery
Pais-Costa et al. (29)	73	Male	An abscess 7 cm in diameter (Lt lobe)	Laparoscopic left hepatectomy	–	Discharged 5 days after surgery
Sato et al. (28)	80	Male	A multiloculated abscess more than 10 cm in diameter (S2/3)	Left hepatectomy	Failure of antibiotic therapy and abscess drainage	Discharged 30 days after surgery
Our case	84	Female	A multiloculated abscess more than 15 cm in diameter (S6/7)	Right posterior sectionectomy	Failure of antibiotic therapy and abscess drainage	Discharged 15 days after surgery

In conclusion, in large multiloculated abscesses after drainage failure, such as in our case, complete liver abscess resection should be performed to ensure earlier recovery and prevent metastatic disease recurrence or secondary metastatic lesions.

## Data availability statement

The original contributions presented in this study are included in the article/Supplementary material, further inquiries can be directed to the corresponding author.

## Ethics statement

Ethics approval was obtained from the Ethical Review Board for Clinical Studies at Teikyo University Chiba Medical Center. Written informed consent was obtained from the patient for the publication of any potentially identifiable images or data included in this article.

## Author contributions

HS was the major contributor to the writing of the manuscript. HS and HN supervised the manuscript and performed the surgical therapy. TM and MY were involved in surgical therapy and patient care. KY and SS supported the pathological examination results. KS, CK, AU, and KK supported the clinical examinations. All authors have read and approved the final manuscript.

## Supplementary material

Supplementary Figure 1 Percutaneous liver abscess drainage. Percutaneous drainage of the liver abscess was performed twice. The additional drainage of the liver abscess with two tubes (white arrowhead) was carried out following drainage with three tubes (black arrowhead).

Supplementary Figure 2 Clinical course of the present case. The patient still had low-grade fever, occasionally 38°C, accompanied by elevated C-reactive protein (CRP) levels.

Supplementary Figure 3 **(A)** Intraoperative appearance of the abscess surrounded liver parenchyma (white arrowhead). **(B)** Intraoperative view of raw surface of the liver after hepatectomy.

Supplementary Figure 4 Gross image of resected specimen and histopathological microscopy of the liver abscess. **(A)** Resected right posterior section of the liver, revealing the multiloculated and necrotic liver abscess. **(B)** Pyogenic liver abscess with areas of necrosis and inflammatory granulation, composed of a chronic inflammatory infiltrate consisting of lymphocytes, epithelioid macrophages, eosinophils, and neutrophils. The adjacent hepatocytes appeared atrophic.
