# Supplementary figures and images for: Successful hepatic resection for invasive Klebsiella pneumoniae large multiloculated liver abscesses with percutaneous drainage failure: A case report
Source: Front Med (Lausanne). 2023 Jan 6;9:1092879. doi: 10.3389/fmed.2022.1092879 (PMC9852881; doi:10.3389/fmed.2022.1092879)

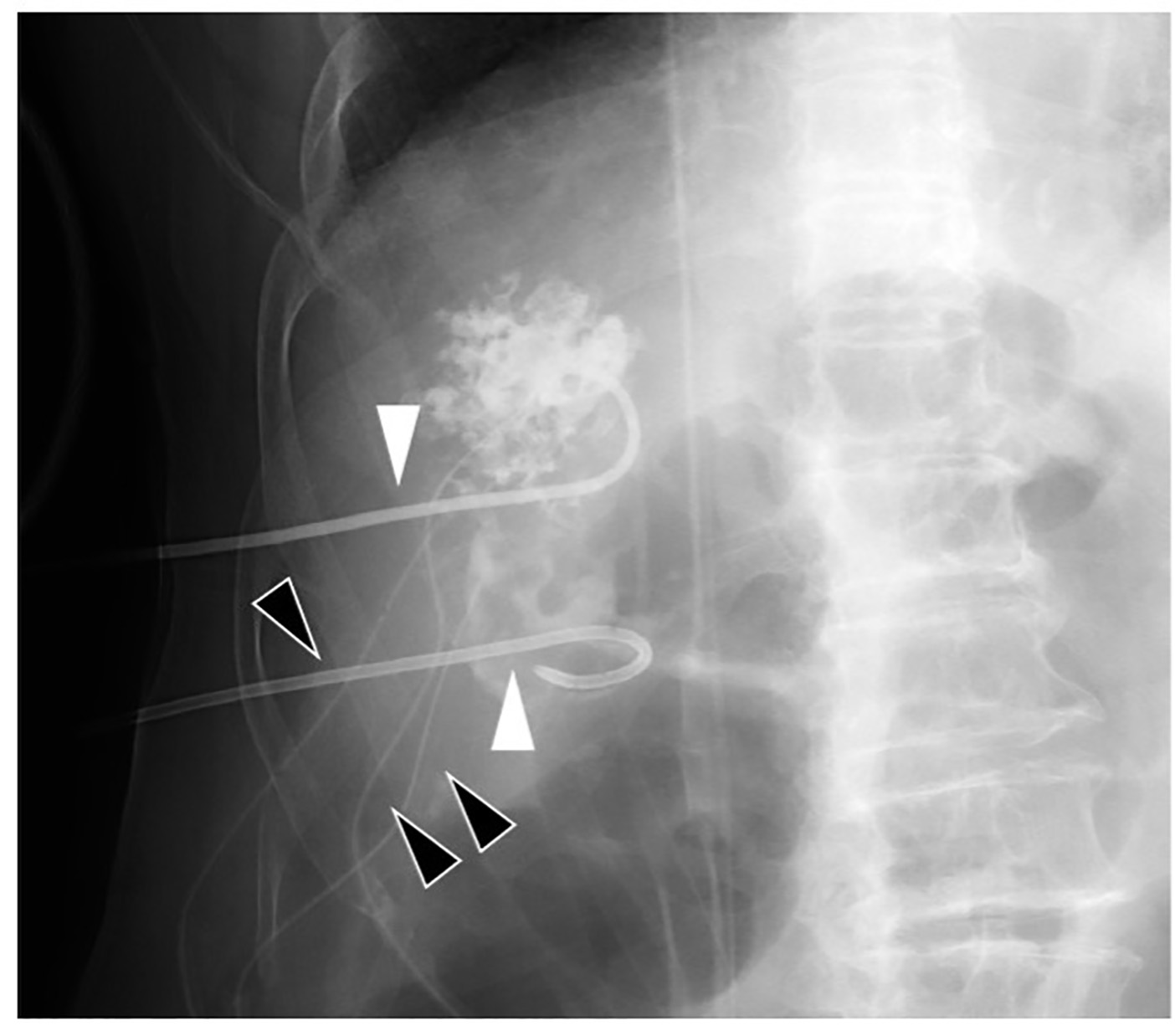

Supplement: Supplementary Figure 1 — Percutaneous liver abscess drainage. Percutaneous drainage of the liver abscess was performed twice. The additional drainage of the liver abscess with two tubes (white arrowhead) was carried out following drainage with three tubes (black arrowhead). [file Image_1.JPEG]

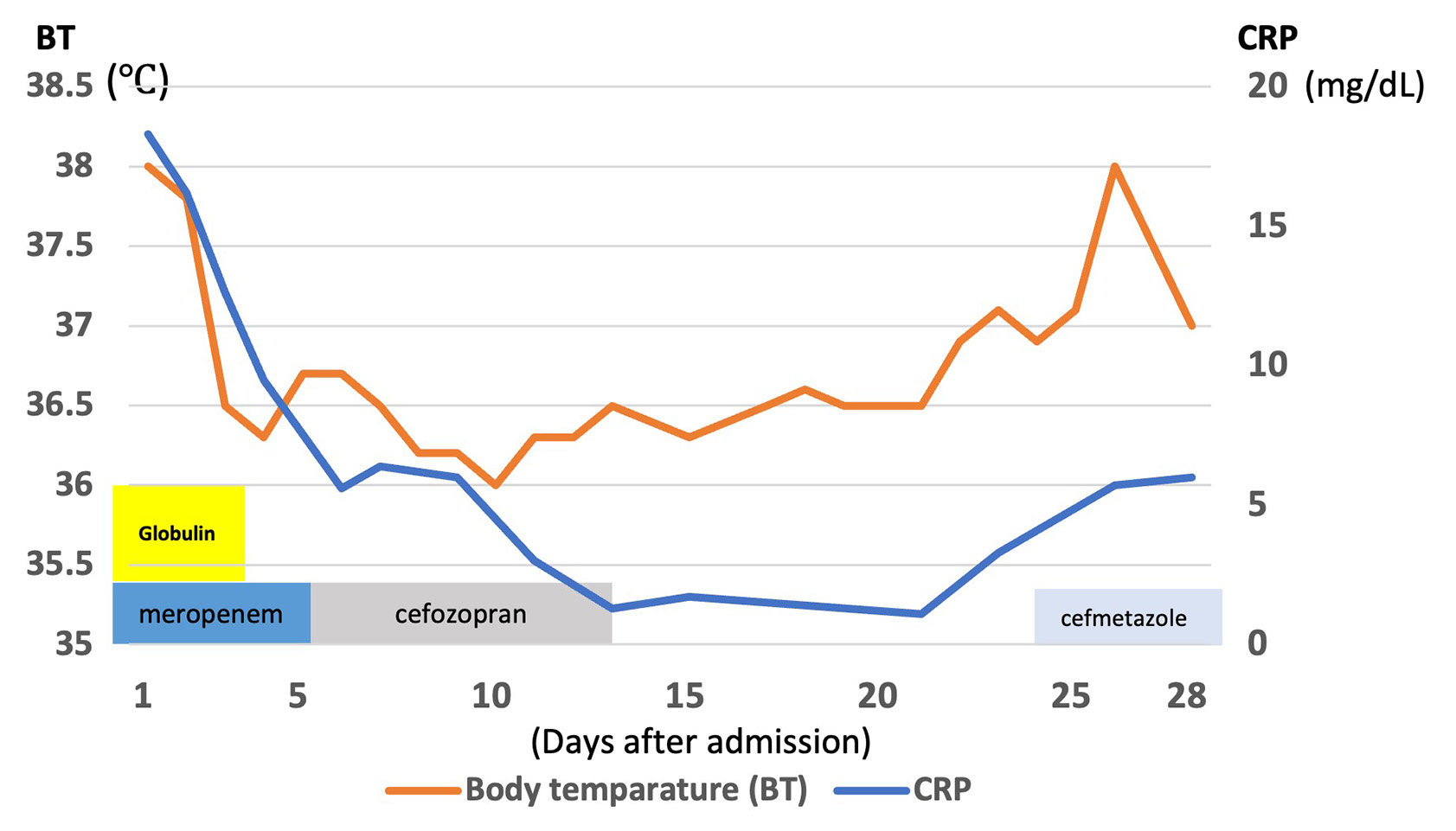

Supplement: Supplementary Figure 2 — Clinical course of the present case. The patient still had low-grade fever, occasionally 38°C, accompanied by elevated C-reactive protein (CRP) levels. [file Image_2.JPEG]

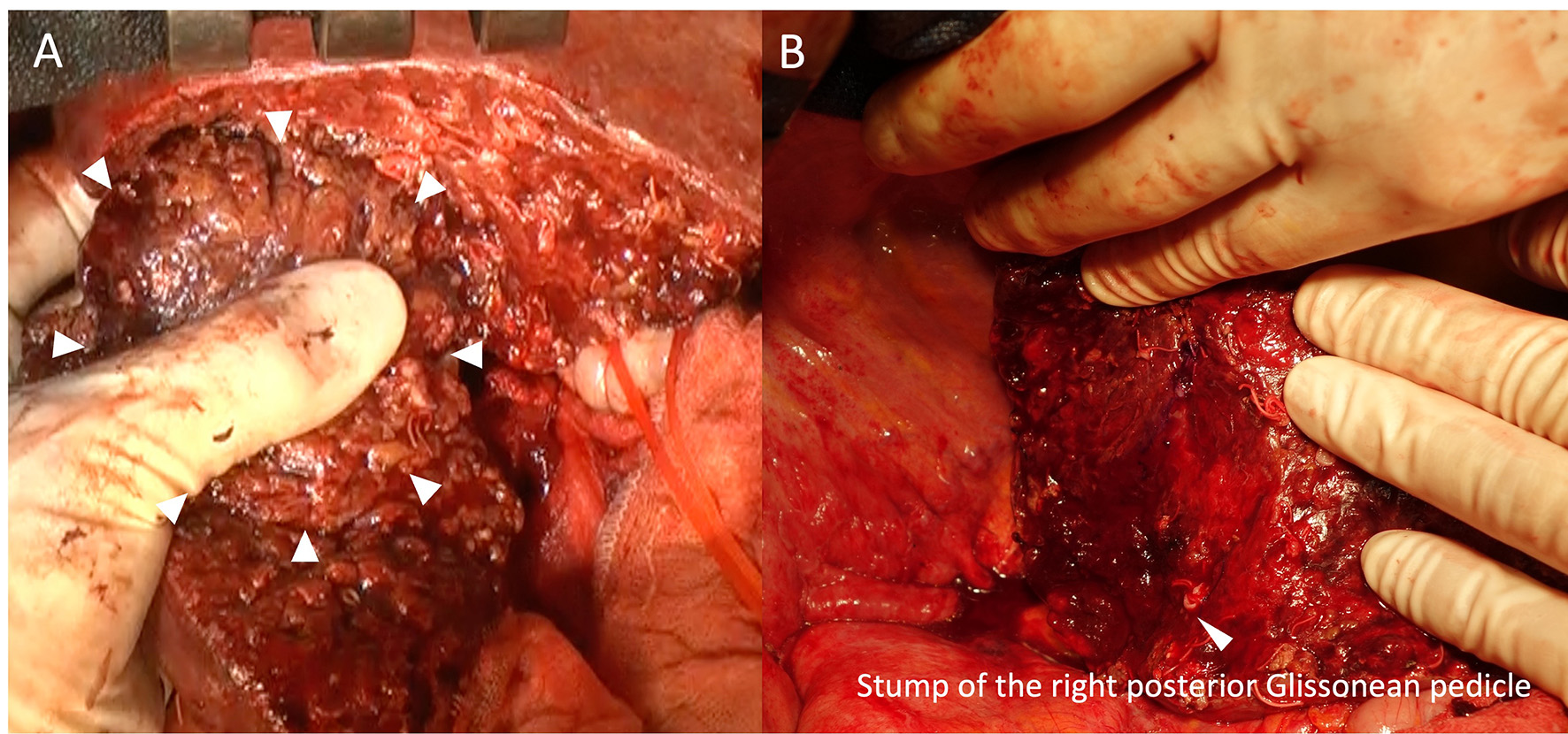

Supplement: Supplementary Figure 3 — (A) Intraoperative appearance of the abscess surrounded liver parenchyma (white arrowhead). (B) Intraoperative view of raw surface of the liver after hepatectomy. [file Image_3.JPEG]

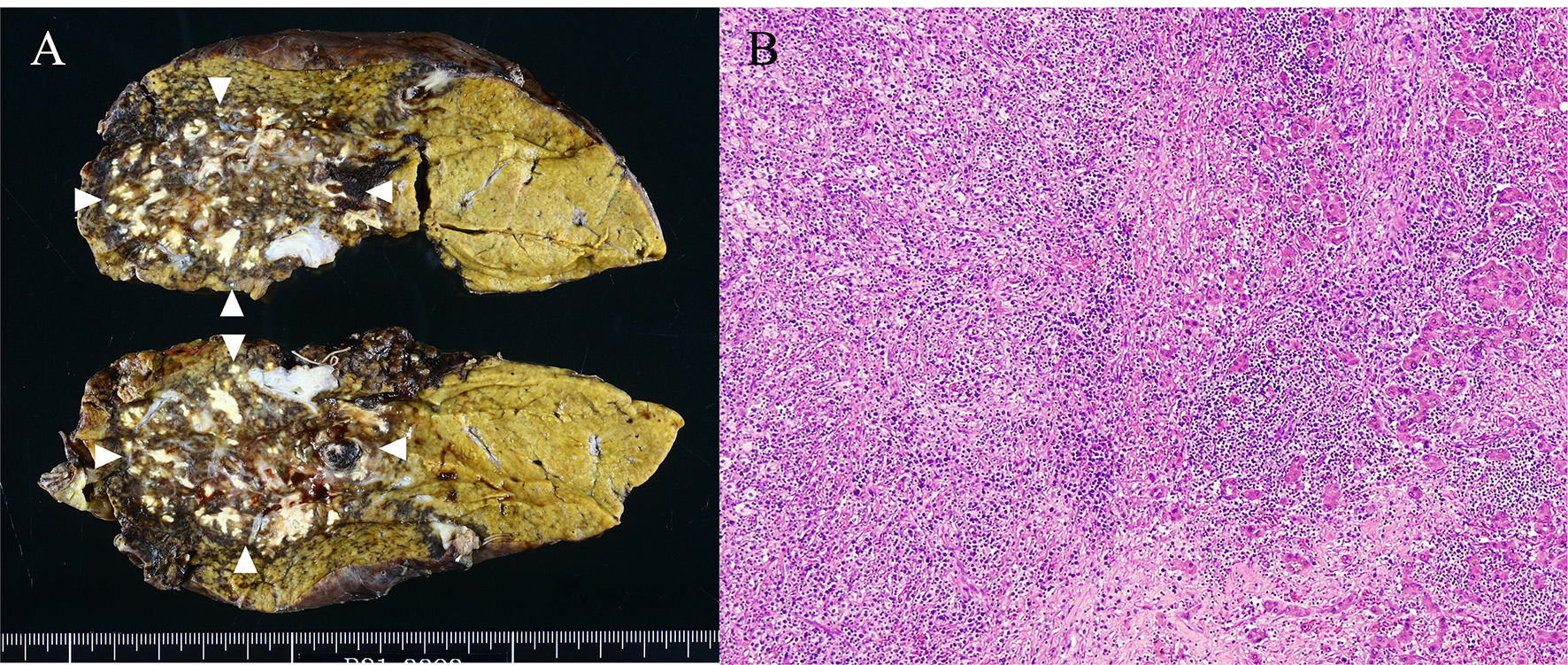

Supplement: Supplementary Figure 4 — Gross image of resected specimen and histopathological microscopy of the liver abscess. (A) Resected right posterior section of the liver, revealing the multiloculated and necrotic liver abscess. (B) Pyogenic liver abscess with areas of necrosis and inflammatory granulation, composed of a chronic inflammatory infiltrate consisting of lymphocytes, epithelioid macrophages, eosinophils, and neutrophils. The adjacent hepatocytes appeared atrophic. [file Image_4.JPEG]
